# Sequential blastocyst transfer improved pregnancy outcomes in patients undergoing frozen-thawed embryo transfer cycles: who will be the ultimate beneficiary

**DOI:** 10.3389/fendo.2026.1782118

**Published:** 2026-09-24

**Authors:** Yanting Cui, Hui Gao, Qingkai Wang, Bin Zhang, Dandan Liu, Huijuan Wei, Qingqing Cheng, Jing Wang, Xiufeng Chang, Kai Deng, Weimin Yang, Liyi Cai

**Affiliations:** 1 Department of Reproductive Medicine, Hebei Maternity Hospital, Shijiazhuang, Hebei, China; 2 Shijiazhuang Birth Defects Precision Prevention and Control Technology Innovation Center, Shijiazhuang, Hebei, China

**Keywords:** blastocyst transfer, frozen-thawed embryo transfer (FET), pregnancy outcomes, sequential embryo transfer, window of implantation (WOI)

## Abstract

**Background:**

Sequential embryo transfer (ET) is a recommended strategy for women with recurrent implantation failure (RIF), but it remains unclear which population may benefit from this strategy. The purpose of this study was to assess the value of sequential blastocyst transfer on pregnancy outcomes in frozen-thawed embryo transfer (FET) cycles and the indications for sequential blastocyst transfer.

**Methods:**

A total of 639 FET cycles were included in this retrospective study, of which 319 were in the conventional group and 320 were in the sequential group. After propensity score matching (PSM), there were 272 FET cycles in each group. Further stratified analyses were conducted based on age, number of previous failed cycles, and number of high-quality blastocysts. Pregnancy outcomes, such as clinical pregnancy rate (CPR), implantation rate, live birth rate (LBR), biochemical pregnancy loss rate, early miscarriage rate, ectopic pregnancy rate, multiple pregnancy rate, and rates of preterm birth and low birthweight in both singleton and multiple pregnancies, were compared between the two groups.

**Results:**

Compared to the conventional group, the sequential group had a higher CPR (65.4% vs. 54.4%, *p* = 0.009) and a lower multiple pregnancy rate (30.9% vs. 45.9%, *p* = 0.005). Stratified analyses revealed that, compared with the conventional group, the sequential group achieved a higher CPR (57.7% vs. 31.7%, *p =* 0.036) and implantation rate (36.5% vs. 20.7%, *p =* 0.044) among patients aged ≥ 40 years (exploratory analysis) and a lower multiple pregnancy rate (31.9% vs. 51.0%, *p =* 0.004) in patients aged < 35 years. Sequential blastocyst transfer was associated with a lower multiple pregnancy rate (27.8% vs. 48.8%, *p =* 0.036) and a higher LBR (58.4% vs. 41.7%, *p =* 0.041) in patients with 3 previous failed cycles, and a higher CPR (63.3% vs. 40.9%, *p =* 0.031) in patients with > 3 previous failed cycles. Additionally, for patients with 0 high-quality blastocysts, the sequential group yielded a higher CPR (54.5% vs. 39.8%, *p =* 0.032) and implantation rate (34.7% vs. 25.2%, *p =* 0.033) than the conventional group. All subgroup analyses are exploratory due to limited statistical power and multiple comparisons.

**Conclusions:**

Patients with ≥ 3 previous failed cycles or 0 high-quality blastocysts, or patients aged ≥ 40 years, may benefit from the sequential blastocyst transfer strategy.

## Introduction

Among individuals aged 15–49 years, the prevalence of infertility is increasing, leading to a persistent global reproductive health issue (1). Since the advent of *in vitro* fertilization (IVF), *in vitro* fertilization and embryo transfer (IVF-ET) have become primary treatments for infertility. To date, 10–13 million babies have been born through this technology, with the actual number potentially being higher (2, 3). However, recurrent implantation failure (RIF) still accounts for 10%–15% of IVF cases (4), resulting in a tremendous psychological and financial burden on patients.

RIF, characterized by the absence of implantation after several embryo transfers, is a clinical phenomenon with no commonly used definition. Factors affecting embryo implantation include embryo quality, endometrial receptivity, embryo–endometrium crosstalk, and the cellular dynamics of the endometrium (5). Therefore, a new transplantation procedure that improves endometrial receptivity and enhances the crosstalk between the embryo and the endometrium was needed to improve clinical pregnancy outcomes in patients with RIF.

Recently, in order to improve pregnancy outcomes in individuals with RIF, researchers in reproductive medicine have developed a sequential transfer strategy (6). Briefly, after endometrial preparation, one cleavage-stage embryo was transferred on day 3, followed by one blastocyst on day 5. Studies involving mice demonstrated that embryo transfer (ET) can enhance endometrial receptivity (7). In humans, sequential ET has positive effects on both implantation and pregnancy compared with conventional ET (6, 8–10). It is well known that suboptimal endometrial receptivity is the most critical factor in RIF (11). Therefore, mechanical stimulation caused by catheter insertion and cytokines produced by the endometrium during the first transfer of the sequential transfer procedure, along with a subsequently more favorable environment for the survival of the subsequently transferred embryo, may be possible reasons for the improved pregnancy outcomes with the sequential transfer strategy (12, 13).

Studies revealed that the prevalence of a displaced window of implantation (WOI) among patients with RIF is 34% (14), and precisely timed ET based on an endometrial receptivity assay may improve clinical pregnancy outcomes in RIF patients (15). Another key factor influencing embryo implantation is the quality and developmental potential of the embryo (16). Embryonic development is regulated by maternal genes during the early stages of oocyte division, and activation of the embryonic genome usually occurs after cell cleavage. Hence, prolonging the *in vitro* culture time before blastocyst transfer is a reliable method to eliminate embryos with low developmental potential and may result in higher embryo implantation rates and pregnancy rates (17).

On this basis, this study modified the traditional sequential transfer strategy. Briefly, after progesterone administration, a blastocyst was transferred on day 5, followed by the transfer of an additional blastocyst on day 6. Compared to the conventional ET strategy, the sequential blastocyst transfer strategy used in this study may offer more advantages, including higher implantation potential, better accommodation of interindividual variations in the WOI, and better adaptability to specific groups. A previous study discovered that sequential blastocyst transfer may improve pregnancy outcomes compared to D3/D5 sequential transfer and double blastocyst transfer (18). In the present study, by classifying participants according to age, the number of previous failed cycles, and the number of high-quality blastocysts, we conducted a detailed analysis of the effect of sequential blastocyst transfer on pregnancy outcomes and provided preliminary evidence regarding patients who may be suitable for this strategy.

## Materials and methods

### Patients and study design

This retrospective study recruited 702 patients at Hebei Maternity Hospital from May 2018 to December 2023. The inclusion criterion was patients undergoing frozen-thawed embryo transfer (FET) with ≥ 2 previous failed ET cycles (including fresh embryo transfer and frozen embryo transfer). The exclusion criteria were as follows: chromosomal abnormalities in female or male patients, uterine malformations, intrauterine adhesions, endometrial tuberculosis, severe endometriosis or adenomyosis, endocrine disease, or autoimmune diseases. Among the 702 patients, 10 were excluded because of chromosomal abnormalities, 22 because of uterine malformations, 17 due to intrauterine adhesions, 1 owing to endometrial tuberculosis, 5 because of severe endometriosis or adenomyosis, and 8 due to endocrine or autoimmune diseases. Finally, a total of 639 patients were enrolled in this study, including the conventional group (n = 319) and the sequential group (n = 320). To reduce selection bias and confounding between the sequential group and the conventional group, we used propensity score matching (PSM) at a ratio of 1:1 using the nearest neighbor method (caliper = 0.05). Variables used to calculate the propensity score included age, body mass index (BMI), infertility duration, anti-Müllerian hormone (AMH), basal follicle-stimulating hormone (bFSH), previous failed cycles, number of miscarriages, primary infertility rate, causes of infertility, endometrial preparation, endometrial thickness, high-quality blastocyst rate, and day 5 blastocyst rate (**Figure 1**). After PSM, standardized mean differences (SMDs) were used to evaluate the matching effectiveness of all covariates. Before matching, notable imbalances were observed in AMH (0.113), previous failed cycles (0.241), hormone replacement therapy (HRT) cycle (0.127), stimulation cycle (0.147), high-quality blastocyst rate (0.138), and day 5 blastocyst rate (0.163). After matching, all SMD values decreased to below the balance threshold of 0.1; therefore, the data after PSM could be used for subsequent treatment effect assessment. The balance plot is shown in **Supplementary Figure S1**.

**Figure 1:**
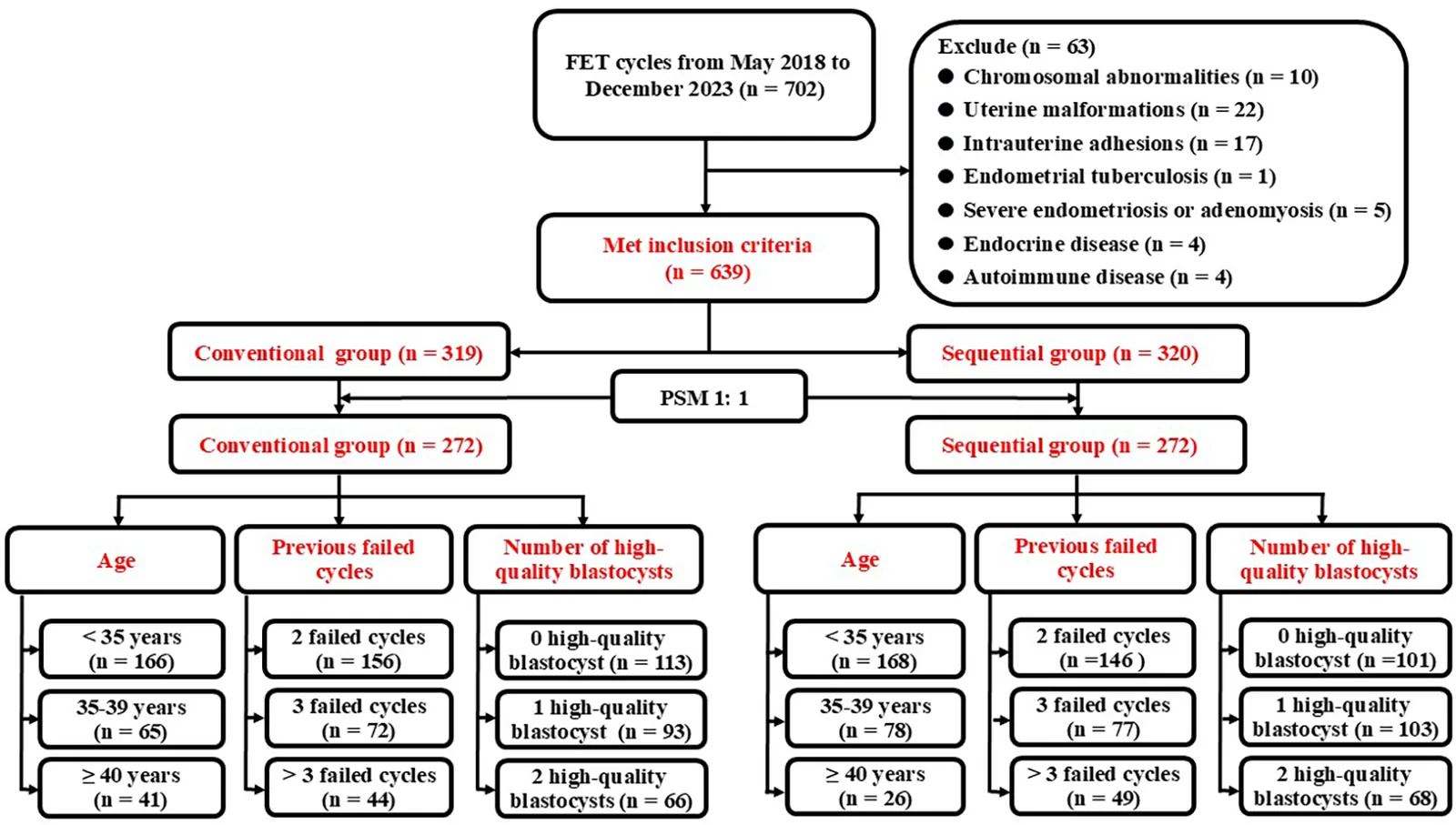
Flow chart of case selection. FET, frozen-thawed embryo transfer; PSM, propensity score matching.

Patients were stratified by age into three subgroups: < 35 years (166 in the conventional group and 168 in the sequential group), 35–39 years (65 in the conventional group and 78 in the sequential group), and ≥ 40 years (41 in the conventional group and 26 in the sequential group) (19). Patients were categorized according to the number of previous failed cycles: 2 previous failed cycles (156 in the conventional group and 146 in the sequential group), 3 previous failed cycles (72 in the conventional group and 77 in the sequential group), and > 3 previous failed cycles (44 in the conventional group and 49 in the sequential group). Patients were classified based on blastocyst quality into the following groups: 0 high-quality blastocysts (113 in the conventional group and 101 in the sequential group), 1 high-quality blastocyst (93 in the conventional group and 103 in the sequential group), and 2 high-quality blastocysts (66 in the conventional group and 68 in the sequential group).

### Embryo assessment

Blastocyst grading, which mainly involved the degree of expansion, the quality of the inner cell mass (ICM), and the morphology of the trophectoderm (TE), was performed according to the Gardner scoring system (20). Blastocyst expansion was graded from 1 to 6 according to blastocoel size and zona pellucida integrity, and the ICM and TE were graded as A, B, or C based on cellular quantity and organizational structure. In this study, blastocysts with a Gardner score of ≥ 3BB were defined as high-quality blastocysts. Early-stage blastocysts or blastocysts with a Gardner score of CC, regardless of the degree of expansion, were not eligible for transfer. All assessments were performed independently by two experienced embryologists.

### Endometrial preparation and ET

Endometrial preparation protocols included hormone replacement therapy (HRT) cycles, natural cycles, and stimulation cycles. In HRT cycles, estradiol valerate tablets (Progynova, Bayer, Berlin, Germany) were initiated at a dose of 6–8 mg daily on days 2–3 of the menstrual cycle. Then, serum estradiol and progesterone levels were monitored periodically, and endometrial thickness was assessed via transvaginal ultrasonography. Once the endometrial thickness reached ≥ 8 mm, 60 mg/d progesterone (Xianju, Zhejiang, China) was administered for luteal support. In natural cycles, transvaginal ultrasonography was performed from days 11–12 of the menstrual cycle onward to monitor follicular development and endometrial thickness. Progesterone (Xianju, Zhejiang, China) was administered at 40 mg/d on the day of ovulation. In ovarian stimulation cycles, letrozole (Hengrui, Jiangsu, China) was administered on day 3 of the menstrual cycle at a dose of 2.5 mg/d for 5 days. Endometrial thickness and the diameter of the dominant follicle were monitored by transvaginal ultrasonography, and human menopausal gonadotropin (hMG, Lizhu, Guangdong, China) was administered when necessary. Triggering with intramuscular human chorionic gonadotropin (hCG, Lizhu, Guangdong, China) at a dose of 8,000–10,000 IU was performed when at least one follicle exceeded 18 mm in diameter. The remaining procedures were consistent with the natural cycle protocol.

In the conventional group, two blastocysts were simultaneously transferred on day 5 after progesterone administration. In the sequential group, one blastocyst was transferred on day 5, followed by an additional blastocyst on day 6 after progesterone administration.

### Luteal support

After ET procedures, luteal support was administered using 20 mg dydrogesterone (Abbott Biologicals B.V., Duffton, The Netherlands) and 90 mg progesterone vaginal gel (Merck Serono, Snowrone, UK) daily until 9–12 weeks of gestation.

### Baseline characteristics and outcome measurements

Data on age, body mass index (BMI), infertility duration, infertility type, number of previous failed cycles, number of miscarriages, anti-Müllerian hormone (AMH) level, baseline follicle-stimulating hormone (bFSH) level, causes of infertility, endometrial preparation protocols, endometrial thickness at the time of ET, the number of high-quality blastocysts, and day 5 blastocyst rate were recorded.

Pregnancy outcomes mainly included clinical pregnancy rate (CPR), implantation rate, live birth rate (LBR), biochemical pregnancy loss rate, early miscarriage rate, ectopic pregnancy rate, multiple pregnancy rate, and rates of preterm birth and low birthweight in both singleton and multiple pregnancies were compared between the two groups. Clinical pregnancy was defined as the presence of a fetal heartbeat on ultrasonography at 4–5 weeks after ET. The implantation rate was defined as the number of gestational sacs divided by the total number of blastocysts transferred. Live birth rate was defined as the percentage of transfers resulting in a live birth. Biochemical pregnancy loss was defined as a serum β-human chorionic gonadotropin (β-hCG) concentration > 30 mIU/mL at 7 days after ET, followed by a subsequent decline with the absence of an intrauterine gestational sac on transvaginal ultrasound. Early miscarriage was defined as the loss of an intrauterine pregnancy before 10 weeks of gestation. Ectopic pregnancy was defined as an extrauterine pregnancy, confirmed by transvaginal ultrasonography or laparoscopy. Multiple pregnancy was defined as the presence of two or more gestational sacs confirmed by ultrasonography. Preterm birth was defined as a live birth occurring between 28 and 37 weeks. Low birthweight was defined as an infant birth weight < 2,500 g.

### Statistical analysis

SPSS 25.0 (SPSS Inc, Chicago, IL, USA) and GraphPad Prism 9 (GraphPad Software, La Jolla, CA, USA) were used for statistical analyses. A 1:1 PSM with a caliper of 0.05 was used to balance baseline characteristics and improve the comparability between the conventional and sequential groups. Continuous data were expressed as the mean ± standard deviation (SD). Categorical data were presented as numbers (percentages). Continuous data conforming to a normal distribution were compared using an independent *t*-test, and the Mann–Whitney U test was used to calculate *p-*values for non-normally distributed data. The χ2 test was used to compare categorical variables. A *post-hoc* power analysis was used to assess the statistical power. Bootstrapping procedures (10,000 resamplings; 95% CI BCa) were performed to obtain higher reliability of the results for all subgroup odds ratios (ORs). In order to fully assess the impact of blastocyst quality on pregnancy outcomes, both blastocyst 1 and blastocyst 2 were classified into three quality levels: level 1 was defined as a Gardner score of ≥ 4BB for a day 5 blastocyst; level 2 was defined as a Gardner score of ≥ 3BB for a day 6 blastocyst; level 3 was defined as other Gardner scores for day 5 and day 6 blastocysts. Effects of transfer strategies on pregnancy outcomes were evaluated using multivariable binary logistic regression adjusting for ET strategy, previous failed cycles, endometrial preparation, age, causes of infertility, infertility duration, number of miscarriages, bFSH, AMH, blastocyst 1 quality, blastocyst 2 quality, and endometrial thickness, as these variables are known clinical confounders associated with IVF outcomes. Adjusted ORs and 95% confidence intervals (CIs) were reported. A *p-*value less than 0.05 was considered statistically significant.

## Results

### Clinical and cycle characteristics

A total of 639 patients met the inclusion criteria, and 544 individuals were included after PSM. Before PSM, the conventional group exhibited a lower number of previous failed cycles (2.63 ± 1.02 vs. 2.92 ± 1.36, *p =* 0.004), a lower high-quality blastocyst rate (37.6% vs. 48.3%, *p <* 0.001), and a lower day 5 blastocyst rate (36.1% vs. 48.4%, *p <* 0.001) compared to the sequential group (**Supplementary Table S1**). After PSM, there were no significant differences in baseline characteristics, which indicated good comparability between the two groups (**Table 1**).

**Table 1:** Clinical and cycle characteristics of the studied groups after PSM.

Variable	Conventional group (n = 272)	Sequential group (n = 272)	*p*-value
Age (years)	33.69 ± 4.95	33.65 ± 4.36	0.864
BMI (kg/m^2^)	24.10 ± 4.38	24.15 ± 3.97	0.751
Infertility duration (years)	4.35 ± 3.03	4.40 ± 2.86	0.623
AMH (ng/mL)	4.03 ± 3.81	4.31 ± 3.79	0.408
bFSH (mIU/mL)	7.66 ± 2.81	7.51 ± 2.56	0.531
Previous failed cycles (n)	2.69 ± 1.07	2.76 ± 1.11	0.391
Number of miscarriages (n)	0.31 ± 0.76	0.33 ± 0.65	0.456
Primary infertility rate (n, %)	150 (55.1%)	149 (54.8%)	0.931
Causes of infertility (n, %)			0.883
Tube and pelvic factors	158 (58.1%)	167 (61.4%)	
Ovulatory factors	32 (11.8%)	25 (9.2%)	
Male factors	31 (11.4%)	30 (11.0%)	
Couple factors	12 (4.4%)	11 (4.0%)	
Others	39 (14.3%)	39 (14.3%)	
Endometrial preparation (n, %)			0.937
HRT cycle	229 (84.2%)	232 (85.3%)	
Natural cycle	26 (9.6%)	24 (8.8%)	
Stimulation cycle	17 (6.3%)	16 (5.9%)	
Endometrial thickness (mm)	9.65 ± 1.86	9.63 ± 1.84	0.805
High-quality blastocyst rate (n, %)	225/544 (41.4%)	239/544 (43.9%)	0.391
Day 5 blastocyst rate (n, %)	213/544 (39.2%)	234/544 (43.0%)	0.196

AMH, anti-Müllerian hormone; bFSH, basal follicle-stimulating hormone; BMI, body mass index; HRT, hormone replacement therapy; PSM, propensity score matching. Values are shown as the mean ± SD or numbers (%).

### Pregnancy outcomes of patients in the two groups

The sequential group was associated with a higher CPR (65.4% vs. 54.4%, *p =* 0.009), a higher low birthweight rate in twin pregnancies (48.4% vs. 31.0%, *p =* 0.030), and a lower multiple pregnancy rate (30.9% vs. 45.9%, *p =* 0.005) than the conventional group. There were no significant differences in other pregnancy outcomes between the two groups (**Table 2**).

**Table 2:** Pregnancy outcomes of the two groups after PSM.

Variable	Conventional group (n = 272)	Sequential group (n = 272)	*p*-value
Biochemical pregnancy loss rate (%)	40 (14.7%)	35 (12.9%)	0.534
CPR (%)	148 (54.4%)	178 (65.4%)	**0.009**
Implantation rate (%)	215/544 (39.5%)	234/544 (43.0%)	0.242
Multiple pregnancy rate (%)	68/148 (45.9%)	55/178 (30.9%)	**0.005**
Ectopic pregnancy rate (%)	1/148 (0.7%)	1/178 (0.6%)	1.000
Early miscarriage rate (%)	23/148 (15.5%)	34/178 (19.1%)	0.399
LBR (%)	120 (44.1%)	137 (50.4%)	0.144
Singleton			
Preterm birth rate (%)	7/78 (9.0%)	10/105 (9.5%)	0.899
Low birthweight (%)	2/78 (2.6%)	5/105 (4.8%)	0.706
Twin			
Preterm birth rate (%)	19/42 (45.2%)	19/32 (59.4%)	0.228
Low birthweight (%)	26/84 (31.0%)	31/64 (48.4%)	**0.030**

CPR, clinical pregnancy rate; LBR, live birth rate; PSM, propensity score matching. Values are shown as numbers (%). Bold values are statistically significant.

### Comparison of pregnancy outcomes between the two groups based on age

Comparisons of pregnancy outcomes between the sequential group and the conventional group based on age were depicted in detail in **Supplementary Table S2**. In the exploratory analysis of patients aged ≥ 40 years, the sequential group was associated with a numerically higher CPR (57.7% vs. 31.7%, *p* = 0.036; post-hoc power = 56%) and implantation rate (36.5% vs. 20.7%, *p* = 0.044; post-hoc power = 52%) than the conventional group (**Figures 2A, B**), but these findings are limited by the small sample size and low statistical power and require validation in larger studies. Among patients aged < 35 years, the sequential group achieved a lower multiple pregnancy rate (31.9% vs. 51.0%, *p =* 0.004; post-hoc power = 81%) (**Figure 2C**). The sequential group had a higher low birthweight rate in twin pregnancies (78.6% vs. 30.0%, *p* = 0.050; post-hoc power = 66%) compared to the conventional group in patients aged 35–39 years (**Figure 2D**), and this was also an exploratory analysis. No significant differences were observed in other pregnancy outcomes between the two groups.

**Figure 2:**
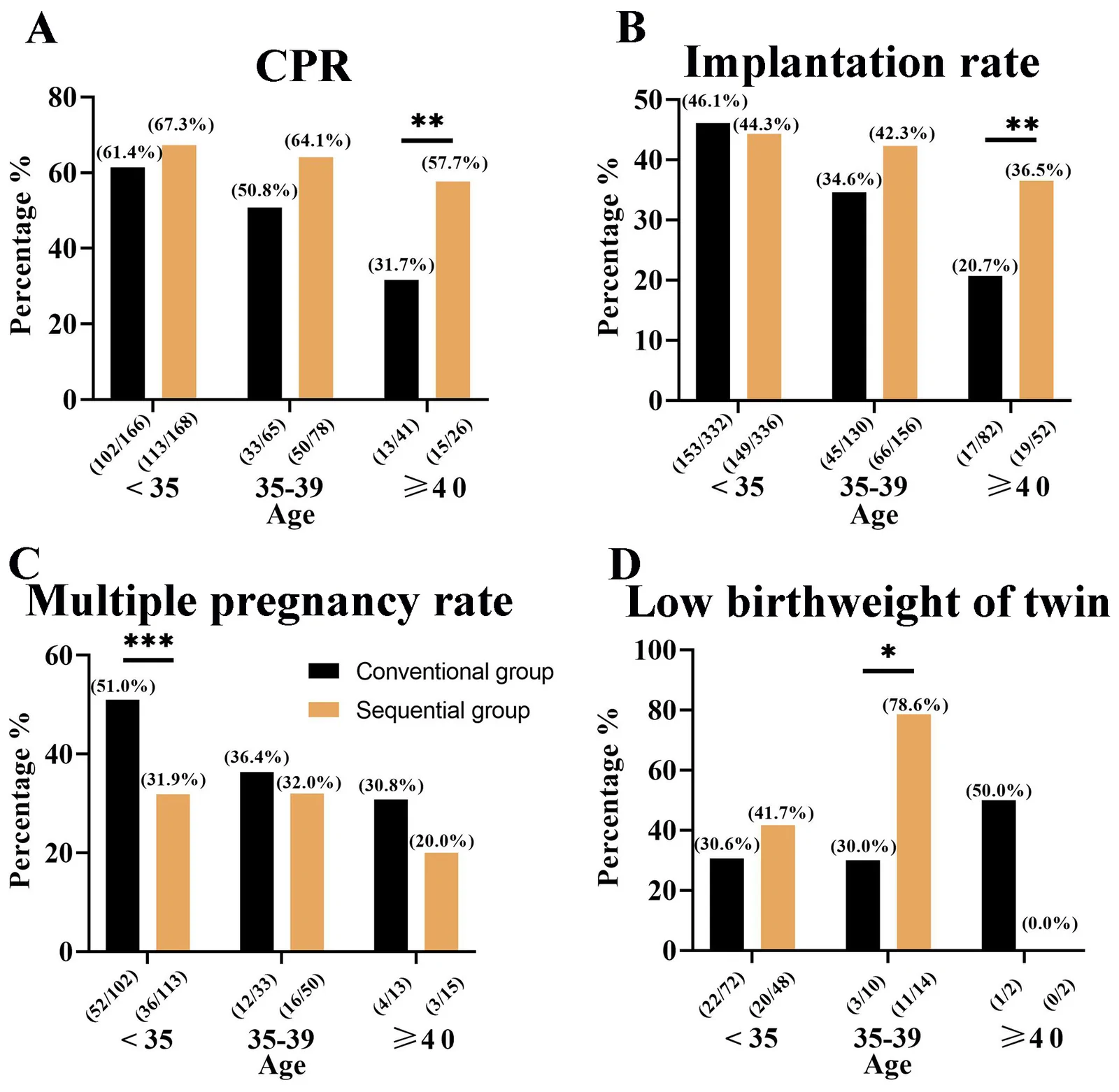
Pregnancy outcomes of patients in the sequential group and conventional group across different age groups. **(A, B)** Sequential blastocyst transfer improved the CPR and implantation rate of patients aged ≥ 40 years; **(C)** For patients aged < 35 years, sequential blastocyst transfer reduced the multiple pregnancy rate; **(D)** For patients aged 35–39 years, sequential blastocyst transfer resulted in a numerically higher but not statistically significant low birthweight rate in twin pregnancies. CPR, clinical pregnancy rate. *p < 0.1; **p < 0.05; ***p < 0.01.

### Comparison of pregnancy outcomes between the two groups based on the number of previous failed cycles

The sequential group obtained a higher CPR (63.3% vs. 40.9%, *p =* 0.031; post-hoc power = 58%) and LBR (49.0% vs. 29.5%, *p =* 0.056; post-hoc power = 48%) compared to the conventional group in patients with > 3 previous failed cycles (**Figures 3A, D**). For patients with 3 previous failed cycles, the sequential group showed a higher CPR (70.1% vs. 56.9%, *p =* 0.094; post-hoc power = 39%) and LBR (58.4% vs. 41.7%, *p =* 0.041; post-hoc power = 53%), and a lower multiple pregnancy rate (27.8% vs. 48.8%, *p =* 0.036; post-hoc power = 56%) compared to the conventional group (**Figures 3A, B, D**). For patients with 2 previous failed cycles, the sequential group exhibited a higher early miscarriage rate (20.4% vs. 11.2%, *p* = 0.090; post-hoc power = 40%) (**Figure 3C**). These results were all exploratory analyses, and there were no significant differences in the remaining pregnancy outcomes between the two groups (**Supplementary Table S3**).

**Figure 3:**
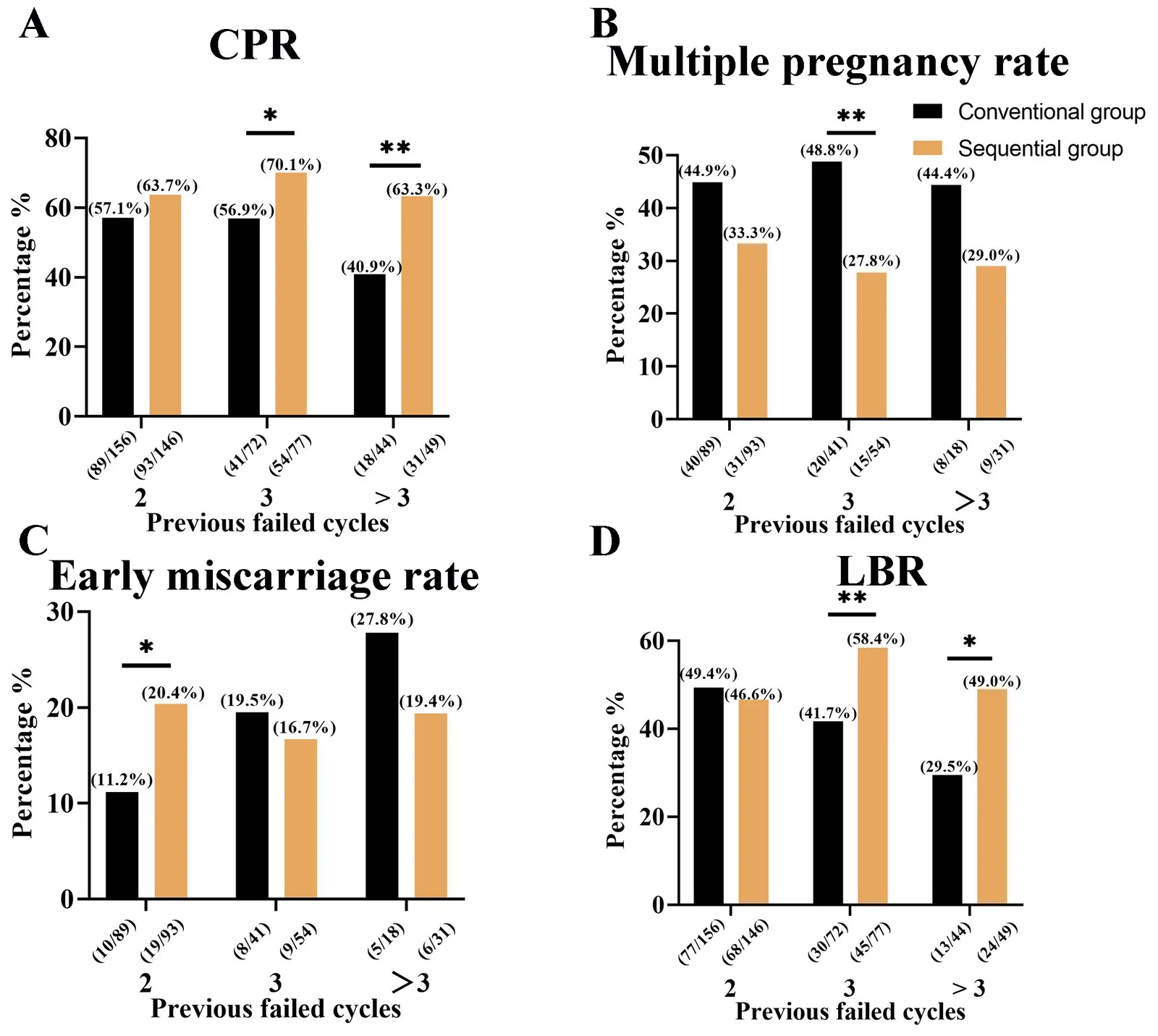
Sequential blastocyst transfer was effective in improving pregnancy outcomes in patients with different numbers of previous failed cycles. **(A)** Sequential blastocyst transfer improved the CPR of patients with > 3 previous failed cycles and resulted in a numerically higher but not statistically significant CPR in patients with 3 previous failed cycles; **(B)** Sequential blastocyst transfer reduced the multiple pregnancy rate in patients with 3 previous failed cycles; **(C)** For patients with two previous failed cycles, sequential blastocyst transfer resulted in a numerically lower but not statistically significant increased early miscarriage rate; **(D)** Sequential blastocyst transfer improved the LBR in patients with 3 previous failed cycles and resulted in a numerically higher but not statistically significant LBR in patients with > 3 previous failed cycles. CPR, clinical pregnancy rate; LBR, live birth rate. *p < 0.1; **p < 0.05.

### Comparison of pregnancy outcomes between the two groups based on the number of high-quality blastocysts

In an exploratory analysis of patients with 0 high-quality blastocysts, the sequential group obtained a higher CPR (54.5% vs. 39.8%, *p =* 0.032; post-hoc power = 57%) and implantation rate (34.7% vs. 25.2%, *p =* 0.033; post-hoc power = 57%) than the conventional group (**Figures 4A, B**). **Figure 4A** showed that there was a higher CPR (67.0% vs. 53.8%, *p =* 0.058; post-hoc power = 47%) in the sequential group than in the conventional group in patients with 1 high-quality blastocyst (exploratory analysis). Additionally, the sequential group exhibited a lower implantation rate (54.4% vs. 66.7%, *p =* 0.040; post-hoc power = 54%; exploratory analysis) and multiple pregnancy rate (37.0% vs. 66.0%, *p =* 0.003; post-hoc power = 85%) than the conventional group in patients with 2 high-quality blastocysts (**Figures 4B, C**). No significant differences were observed in other pregnancy outcomes between the two groups (**Supplementary Table S4**).

**Figure 4:**
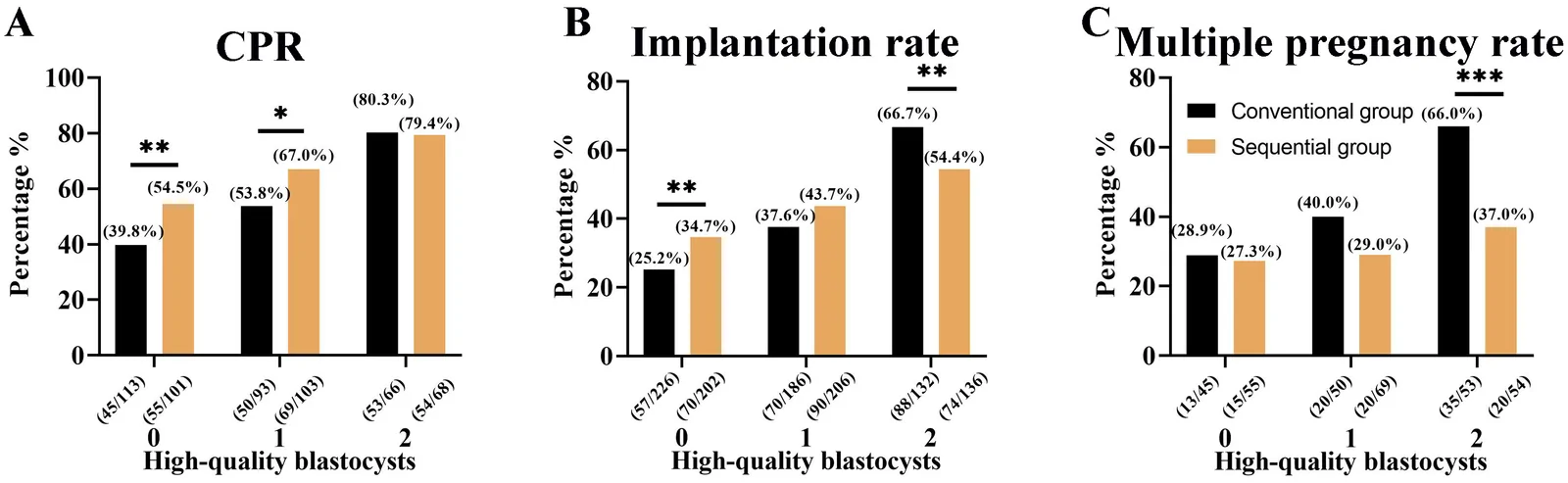
Sequential blastocyst transfer had multiple effects on pregnancy outcomes in patients with different numbers of high-quality blastocysts. **(A)** Sequential blastocyst transfer improved the CPR of patients with 0 high-quality blastocysts and numerically higher but not statistically significant improved CPR in patients with 1 high-quality blastocyst; **(B)** Sequential blastocyst transfer improved the implantation rate in patients with 0 high-quality blastocysts and decreased the implantation rate in patients with 2 high-quality blastocysts; **(C)** For patients with 2 high-quality blastocysts, sequential blastocyst transfer reduced the multiple pregnancy rate. CPR, clinical pregnancy rate. *p < 0.1; **p < 0.05; ***p < 0.01.

### Effects of ET strategies on pregnancy outcomes

ET strategies, previous failed cycles, endometrial preparation protocols, age, causes of infertility, infertility duration and type, number of miscarriages, bFSH level, AMH level, blastocyst 1 quality, blastocyst 2 quality, and endometrial thickness were included in the binary logistic regression analysis to evaluate the effectiveness of ET strategies on pregnancy outcomes (**Table 3**). Compared to the conventional group, the sequential group had a significantly higher CPR (OR 1.524, 95% CI 1.048–2.217, *p* = 0.028). CPR was also associated with blastocyst 1 quality (level 3 vs. level 1, OR 0.576, 95% CI 0.357–0.928, *p* = 0.024) and blastocyst 2 quality (level 3 vs. level 1, OR 0.464, 95% CI 0.248–0.866, *p* = 0.016). For causes of infertility, male factors had a higher CPR than tubal and pelvic factors (OR 1.932, 95% CI 1.015–3.676, *p =* 0.045). Blastocyst 2 quality (level 3 vs. level 1, OR 0.259, 95% CI 0.131–0.513, *p* **<** 0.001) was negatively associated with multiple pregnancy. Compared to HRT cycles, the use of natural cycles for endometrial preparation was associated with a lower multiple pregnancy rate (OR 0.127, 95% CI 0.030–0.547, *p =* 0.006). LBR was associated with age (OR 0.932, 95% CI 0.891–0.976, *p =* 0.003), blastocyst 1 quality (level 3 vs. level 1, OR 0.621, 95% CI 0.389–0.992, *p* = 0.046), and blastocyst 2 quality (level 3 vs. level 1, OR 0.394, 95% CI 0.218–0.712, *p* **=** 0.002).

**Table 3:** Binary logistic regression analysis of possible correlates of pregnancy outcomes after PSM.

Variable	CPR		Multiple pregnancy		LBR	
	Adjusted OR (95% CI)	*p*-value	Adjusted OR (95% CI)	*p*-value	Adjusted OR (95% CI)	*p*-value
ET strategies						
Conventional ET	Ref		Ref		Ref	
Sequential blastocyst transfer	1.524 (1.048–2.217)	0.028	0.747 (0.477–1.171)	0.204	1.310 (0.907–1.893)	0.150
Previous failed cycles	0.939 (0.789–1.116)	0.474	0.928 (0.728–1.183)	0.545	0.924 (0.773–1.105)	0.386
Endometrial preparation						
HRT cycle	Ref		Ref		Ref	
Natural cycle	0.562 (0.298–1.058)	0.074	0.127 (0.030–0.547)	0.006	0.703 (0.371–1.333)	0.281
Stimulation cycle	0.833 (0.380–1.827)	0.649	0.563 (0.211–1.498)	0.250	0.795 (0.367–1.726)	0.562
Female age	0.961 (0.918–1.005)	0.083	0.967 (0.915–1.021)	0.223	0.932 (0.891–0.976)	0.003
Causes of infertility						
Tube and pelvic factors	Ref		Ref		Ref	
Ovulatory factors	1.656 (0.814–3.369)	0.164	1.889 (0.865–4.128)	0.111	1.789 (0.909–3.521)	0.092
Male factors	1.932 (1.015–3.676)	0.045	1.009 (0.479–2.126)	0.981	1.494 (0.817–2.731)	0.193
Couple factors	1.377 (0.543–3.495)	0.501	0.282 (0.060–1.325)	0.109	0.992 (0.400–2.459)	0.987
Others	1.140 (0.663–1.961)	0.635	1.053 (0.544–2.040)	0.878	1.018 (0.592–1.751)	0.948
Infertility type						
Primary	Ref		Ref		Ref	
Secondary	1.095 (0.701–1.710)	0.690	0.720 (0.423–1.225)	0.225	0.929 (0.599–1.438)	0.740
Infertility duration	0.981 (0.917–1.050)	0.580	0.973 (0.895–1.059)	0.529	1.005 (0.940–1.075)	0.887
Number of miscarriages	0.880 (0.655–1.183)	0.398	0.886 (0.599–1.312)	0.547	0.827 (0.599–1.142)	0.249
bFSH	1.012 (0.940–1.090)	0.745	0.977 (0.891–1.072)	0.629	1.043 (0.969–1.122)	0.264
AMH	1.034 (0.973–1.098)	0.278	0.936 (0.873–1.004)	0.065	0.992 (0.937–1.050)	0.791
Blastocyst 1 quality						
Level 1	Ref		Ref		Ref	
Level 2	1.320 (0.732–2.380)	0.356	0.777 (0.403–1.498)	0.451	0.816 (0.469–1.419)	0.472
Level 3	0.576 (0.357–0.928)	0.024	0.588 (0.336–1.030)	0.063	0.621 (0.389–0.992)	0.046
Blastocyst 2 quality						
Level 1	Ref		Ref		Ref	
Level 2	0.640 (0.345–1.185)	0.156	0.711 (0.386–1.312)	0.276	0.583 (0.327–1.039)	0.067
Level 3	0.464 (0.248–0.866)	0.016	0.259 (0.131–0.513)	<0.001	0.394 (0.218–0.712)	0.002
Endometrial thickness (mm)						
<8	Ref		Ref		Ref	
8–12	1.466 (0.873–2.549)	0.176	2.067 (0.927–4.613)	0.076	1.468 (0.827–2.607)	0.189
>12	1.352 (0.630–2.902)	0.438	2.322 (0.829–6.505)	0.109	1.262 (0.580–2.744)	0.558

AMH, anti-Müllerian hormone; bFSH, basal follicle-stimulating hormone; CI, confidence interval; CPR, clinical pregnancy rate; ET, embryo transfer; HRT, hormone replacement therapy; LBR, live birth rate; OR, odds ratio. Bold values are statistically significant.

## Discussion

The pregnancy outcomes of IVF remain uncertain; previous studies reported that the CPR of IVF was 40%–50% (21, 22). Notably, implantation failure remains a major limiting factor. There is a viewpoint that two-thirds of IVF-ET failures are due to a lack of endometrial receptivity and one-third are due to poor embryo quality (23), which emphasizes the importance of endometrial receptivity in the IVF procedure. Currently, embryo culture systems in reproductive laboratories are highly optimized, and improving endometrial receptivity has become a central strategy for improving the overall success rate (24).

Our study showed that the sequential group achieved a higher CPR compared to the conventional group (65.4% vs. 54.4%, *p* = 0.009), which was consistent with previous studies (8, 18, 25). WOI is a transient critical period, during which the endometrium transitions from a non-receptive to a receptive state through complex cellular and molecular changes (26), typically between days 20 and 24 of the 28-day menstrual cycle (27). Previous studies reported that WOI displacement was found in at least 25% of patients with RIF, among whom 84% exhibited a prereceptive endometrial status (28). Thus, sequential blastocyst transfer may enhance the probability of embryoendometrial synchrony by covering a broader receptive period. The synchronization between embryonic development and endometrial receptivity, along with timely bidirectional communication, plays a pivotal role in successful implantation (29, 30). Additionally, the initial transfer of a blastocyst appears to induce endometrial transition, creating a more receptive microenvironment for the subsequent blastocyst (9, 31, 32). A previous study provided a detailed exposition of the uterine changes, inflammatory responses, and immune factors involved in human embryo implantation (5). However, whether sequential transfer affects these processes remains to be explored in future basic research. Compared with that of patients with RIF, the CPRs of both groups in this study were significantly higher. This may be because some of the participants we included had undergone two failed embryo transfer cycles, and the quality of the embryos they received was not included in the inclusion criteria, which differs from the situation for RIF patients. Therefore, among the patients we recruited, there might be those who experienced two failed cycles but still had a normal prognosis, which may lead to the CPR improved.

Our study also observed a lower multiple pregnancy rate in the sequential group compared to the conventional group (30.9% vs. 45.9%, *p* = 0.005). The high multiple pregnancy rate in the conventional group was partly attributable to patient selection. Since the patients had experienced at least two failed cycles, they were more inclined to accept the risks associated with multiple pregnancy rather than the risk of pregnancy failure, even though they were offered the option of selective single blastocyst transfer. After confirmation of CPR, 40 patients underwent fetal reduction (including two triplet pregnancies), of whom, only 6 patients underwent fetal reduction treatment (all in the conventional group), and the remaining patients underwent spontaneous multifetal pregnancy reduction. Regarding the lower multiple pregnancy rate in the sequential group, we hypothesize that the first blastocyst in the sequential protocol may induce endometrial priming, potentially restricting successful implantation of the second blastocyst to a narrower receptive window. However, this mechanism is speculative and limited in the absence of an endometrial receptivity assay (ERA) or histological validation, and should be further validated in future animal histopathological experiments. Our study showed that, compared to the conventional group, the early miscarriage rate in the sequential group increased by 3.6%, which might be a factor that patients need to consider when choosing a transfer strategy. However, there are no studies on the impact of the sequential transfer strategy on early miscarriage, and further research is needed to investigate the relationship between the sequential transfer strategy and early miscarriage. This study indicates that the LBR in the sequential group was not significantly higher than that in the conventional group; the higher early miscarriage rate in the sequential group might also be a possible reason why the increased CPR did not ultimately lead to a higher LBR. Our study also showed that, in the sequential group, the rates of preterm birth and low birthweight in twin pregnancies showed an upward trend, which might impose an additional burden on patients and should be carefully considered. Therefore, considering the increased CPR, the multiple pregnancy rate, the early miscarriage rate, and the rates of preterm birth and low birthweight in twin pregnancies, the applicable population for the sequential transfer strategy and its safety after application still require further exploration.

To further identify the patients most likely to benefit from sequential blastocyst transfer, a stratified analysis was performed. In patients aged < 35 years, the sequential group exhibited a comparable CPR and a significantly lower multiple pregnancy rate than the conventional group, which was in line with the results reported by Li et al. (33). Sequential transfer may not be suitable for patients aged < 35 years, because this strategy failed to significantly improve pregnancy outcomes. Additionally, the infectious morbidity associated with two transcervical blastocyst transfer procedures has not been systematically evaluated in this cohort and warrants prospective safety monitoring; the two separate transfer procedures also imposed a financial burden on patients. With advancing age, CPR and implantation rate decreased in patients aged > 35 years. However, the downward trend was markedly slower in the sequential group. In patients aged ≥ 40 years, the sequential group had higher CPR and implantation rate than the conventional group. This observation offers a novel direction for identifying the optimal candidates for sequential blastocyst transfer and also poses challenges in balancing clinical benefit against operative risks and economic burden during clinical decision-making.

Results of this study demonstrated that among patients with ≥ 3 previous failed cycles, the sequential group yielded higher CPR and LBR compared to the conventional group, which is consistent with a previous study (9). Furthermore, in patients with two previous failed cycles, the sequential group failed to improve CPR and had a higher early miscarriage rate than the conventional group. The uterine curettage performed due to an early miscarriage may cause damage to the endometrium, thereby making the next pregnancy more difficult. Therefore, for patients with ≥ 3 previous failed cycles, sequential procedures may represent an effective treatment strategy (24, 34).

Qi et al. showed that the sequential transfer strategy improved pregnancy outcomes in patients with one or zero high-quality blastocysts (18). In the present study, the results of the comparison of pregnancy outcomes between the sequential group and the conventional group based on blastocyst quality showed that, among patients with 0 high-quality blastocysts, the sequential group achieved a significantly higher CPR and implantation rate than the conventional group. Among patients with two high-quality blastocysts, the two groups demonstrated comparable CPR and LBR, and the conventional group exhibited a higher implantation rate and multiple pregnancy rate. Based on the speculative mechanism of embryo–endometrium synchrony and uterine receptivity window restriction, we speculate that this may be because the sequential transfer strategy improves the implantation of a single blastocyst in the patient while reducing the simultaneous implantation of two blastocysts. Regression analysis revealed that the endometrial preparation protocol and blastocyst quality are the main factors influencing the multiple pregnancy rate. Considering the relatively small sample size for natural cycles and the lack of direct evidence demonstrating their impact on multiple pregnancies, blastocyst quality remains the primary factor influencing the multiple pregnancy rate. This study also showed that age and blastocyst quality remained key independent factors influencing LBR. Therefore, promoting childbearing at an appropriate age and optimizing embryo quality represent the most effective strategies for improving the LBR. However, for patients without available high-quality embryos, the sequential transfer protocol may be considered appropriate.

The strengths of our study include its real-world design, which reflects actual clinical practice, and complete follow-up through live birth. Through stratified analyses based on age, the number of previous failed cycles, and blastocyst quality, this study identified patient subgroups more likely to benefit from sequential blastocyst transfer. However, several limitations should be acknowledged. First, the data analyzed in this study were obtained from a single center; therefore, selection bias cannot be excluded, and multicenter studies are needed to improve the generalizability of the findings. Second, the relatively high multiple pregnancy rate in both groups should be interpreted in the context of the study population, as all included patients had experienced at least two previous failed transfer cycles and underwent transfer of two blastocysts. Third, this study enrolled patients undergoing FET with ≥ 2 previous failed ET cycles without stratifying by failure causes (such as embryo or endometrial factors), which may lead to residual confounding that cannot be adjusted for during PSM. Finally, considering the absence of guideline endorsement from the European Society of Human Reproduction and Embryology (ESHRE), the American Society for Reproductive Medicine (ASRM), or the Chinese Society of Reproductive Medicine (CSRM) regarding sequential blastocyst transfer for RIF, and considering that the *post-hoc* power analysis revealed that some pregnancy outcome indicators in our stratified analysis had a power < 80%, the stratified analyses should be interpreted as exploratory due to limited statistical power and multiple comparisons, and the conclusions presented in this article also require more detailed research for verification.

## Conclusion

In conclusion, sequential blastocyst transfer can improve pregnancy outcomes in patients undergoing FET cycles. This study confirmed the significant finding that patients aged < 35 years or with two high-quality blastocysts had a lower multiple pregnancy rate after receiving sequential blastocyst transfer. Exploratory preliminary outcomes of this study showed that, for patients with three previous failed cycles, sequential transfer was associated with a higher LBR, and a higher CPR and a numerically higher but not statistically significant LBR were observed in the sequential group among patients with > 3 previous failed cycles; sequential blastocyst transfer also yielded a higher CPR and implantation rate in patients with 0 high-quality blastocysts or patients aged ≥ 40 years. Although all subgroup analyses are exploratory due to limited statistical power and multiple comparisons, this study still provides valuable evidence regarding the application of sequential transplantation strategies.

## Data Availability

The raw data supporting the conclusions of this article will be made available by the authors, without undue reservation.
