# Supplementary material for: Sequential blastocyst transfer improved pregnancy outcomes in patients undergoing frozen-thawed embryo transfer cycles: who will be the ultimate beneficiary
Source: Front Endocrinol (Lausanne). 2026 Sep 24;17:1782118. doi: 10.3389/fendo.2026.1782118 (PMC13662746; doi:10.3389/fendo.2026.1782118)
Supplement: Supplementary file 1 [file Supplementaryfile1.docx]

**Sequential Transfer of Blastocysts Improved Pregnancy Outcomes in Patients with Frozen-thawed Embryo Transfer Cycles: Who Will Be the Ultimate Beneficiary**

**Yanting Cui^1*^, Hui Gao^1*^, Qingkai Wang****^1,2^, Bin Zhang^1^, Dandan Liu^1^, Huijuan Wei^1,2^, Qingqing Cheng^1,2^, Jing Wang^1,2^, Xiufeng Chang^1,2^, Kai Deng^1,2^, Weimin Yang^1,2^** ^🖂^**, Liyi Cai^1,2^** ^🖂^

^1^ Reproductive Medicine Department of Hebei Maternity Hospital, Shijiazhuang, Hebei province, China

^2^ Shijiazhuang Technology Innovation Center of Precision Prevention and Control of Birth Defects, Shijiazhuang city, Hebei province, China

**^*^** These authors have contributed equally to this work

^🖂^Corresponding author:

Liyi Cai, Correspondence address: Cai760829@163.com.

Weimin Yang, Correspondence address: 18762807806@163.com.

**Supplementary table 1. Clinical and cycle characteristics of studied groups before PSM.**

| **Variable** | **Conventional group**  **(n = 319)** | **Sequential group**  **(n = 320)** | ***p*-value** |
| --- | --- | --- | --- |
| **Age (year)** | 33.76±4.97 | 33.72±4.24 | 0.875 |
| **BMI (kg/m^2^)** | 24.06±4.32 | 23.99±3.83 | 0.896 |
| **Infertility duration (year)** | 4.29±3.05 | 4.56±2.90 | 0.120 |
| **AMH (ng/ml)** | 3.93±3.69 | 4.35±3.77 | 0.161 |
| **bFSH (mIU/ml)** | 7.65±2.87 | 7.62±2.74 | 0.862 |
| **Previous failed cycles (n)** | 2.63±1.02 | 2.92±1.36 | **0.004** |
| **Number of miscarriages (n)** | 0.34±0.75 | 0.33±0.65 | 0.959 |
| **Primary infertility rate (n, %)** | 137 (42.9%) | 143 (44.7%) | 0.657 |
| **Causes of Infertility (n, %)** |  |  | 0.730 |
| Tube and pelvic factors | 190 (59.6%) | 193 (60.3%) |  |
| Ovulatory factors | 37 (11.6%) | 28 (8.8%) |  |
| Male factors | 37 (11.6%) | 36 (11.3%) |  |
| Couples’ factor | 12 (3.8%) | 16 (5.0%) |  |
| others | 43 (13.5%) | 47 (14.7%) |  |
| **Endometrial preparation (n, %)** |  |  | 0.153 |
| HRT cycle | 259 (81.2%) | 275 (85.9%) |  |
| Natural cycle | 31 (9.7%) | 28 (8.8%) |  |
| Stimulation cycle | 29 (9.1%) | 17 (5.3%) |  |
| **Endometrial thickness (mm)** | 9.51±1.85 | 9.65±1.79 | 0.339 |
| **High-quality blastocyst rate (n, %)** | 240/638 (37.6%) | 309/640 (48.3%) | **<0.001** |
| **Day5 blastocyst rate (n, %)** | 230/638 (36.1%) | 310/640 (48.4%) | **<0.001** |

AMH, anti-Müllerian hormone; bFSH, basal follicle-stimulating hormone; BMI, body mass index; PSM, propensity score matching. Values are shown as mean ± standard deviation or numbers (%). Bold values are statistically significant.

**Supplementary table 2. Comparison of pregnancy outcomes of all patients based on age after PSM.**

|  | **Conventional group**  **(n = 272)** | | | **Sequential group**  **(n = 272)** | | |  |  |  |
| --- | --- | --- | --- | --- | --- | --- | --- | --- | --- |
| **Age** | **< 35**  **(n = 166)** | **35-39**  **(n = 65)** | **≥ 40**  **(n = 41)** | **< 35**  **(n = 168)** | **35-39**  **(n = 78)** | **≥ 40**  **(n = 26)** | ***p*1**  **OR (95% CI)**  BCa 95% CI | ***p*2**  **OR (95% CI)**  BCa 95% CI | ***p*3**  **OR (95% CI)**  BCa 95% CI |
| **Biochemical pregnancy loss rate (%)** | 24 (14.5%) | 8 (12.3%) | 8 (19.5%) | 20 (11.9%) | 12 (15.4%) | 3 (11.5%) | 0.490  0.800 (0.423-1.511)  (0.401-1.534) | 0.597  1.295 (0.495-3.391)  (0.457-4.359) | 0.603  0.538 (0.129-2.248)  (0.125-2.267) |
| **CPR (%)** | 102 (61.4%) | 33 (50.8%) | 13 (31.7%) | 113 (67.3%) | 50 (64.1%) | 15 (57.7%) | 0.267  1.289 (0.823-2.020)  (0.814-2.061) | 0.108  1.732 (0.885-3.388)  (0.852-3.659) | **0.036^b^**  **2.937 (1.060-8.136)**  **(1.000-10.920)** |
| **Implantation rate (%)** | 153/332 (46.1%) | 45/130 (34.6%) | 17/82 (20.7%) | 149/336 (44.3%) | 66/156 (42.3%) | 19/52 (36.5%) | 0.652  0.932 (0.687-1.264)  (0.683-1.267) | 0.184  1.385 (0.856-2.241)  (0.842-2.297) | **0.044^b^**  **2.201 (1.012-4.788)**  (0.952-5.157) |
| **Multiple pregnancy rate (%)** | 52/102 (51.0%) | 12/33 (36.4%) | 4/13 (30.8%) | 36/113 (31.9%) | 16/50 (32.0%) | 3/15 (20.0%) | **0.004^a^**  **0.450 (0.258-0.783)**  **(0.257-0.777)** | 0.681  0.824 (0.326-2.077)  (0.287-2.314) | 0.827  0.563 (0.100-3.168)  (0.094-3.464) |
| **Ectopic pregnancy rate (%)** | 1/102 (1.0%) | 0/33 (0.0%) | 0/13 (0.0%) | 1/113 (0.9%) | 0/50 (0.0%) | 0/15 (0.0%) | 1.000  0.902 (0.056-14.606)  (0.260-3.076) | - | - |
| **Early miscarriage rate (%)** | 9/102 (8.8%) | 8/33 (24.2%) | 6/13 (46.2%) | 17/113 (15.0%) | 10/50 (20.0%) | 7/15 (46.7%) | 0.162  1.830 (0.777-4.310)  (0.757-5.705) | 0.646  0.781 (0.272-2.245)  (0.240-2.615) | 0.978  1.021 (0.230-4.526)  (0.179-5.714) |
| **LBR (%)** | 89 (53.6%) | 25 (38.5%) | 6 (14.6%) | 90 (53.6%) | 39 (50.0%) | 8 (30.8%) | 0.994  0.998 (0.649-1.535)  (0.641-1.540) | 0.167  1.600 (0.820-3.122)  (0.779-3.366) | 0.113  2.593 (0.780-8.621)  (0.617-14.334) |
| **Singleton** | | | | | | | | | |
| **Preterm birth rate (%)** | 6/53 (11.3%) | 1/20 (5.0%) | 0/5 (0.0%) | 7/66 (10.6%) | 3/32 (9.4%) | 0/7 (0.0%) | 0.901  0.929 (0.293-2.952)  (0.219-4.295) | 0.967  1.966 (0.190-20.322)  - | - |
| **Low birthweight (%)** | 2/53 (3.8%) | 0/20 (0.0%) | 0/5 (0.0%) | 4/66 (6.1%) | 1/32 (3.1%) | 0/7 (0.0%) | 0.885  1.645 (0.290-9.348)  (0.295-6.366) | 1.000  1.032 (0.970-1.099)  - | - |
| **Twin** | | | | | | | | | |
| **Preterm birth rate (%)** | 15/36 (41.7%) | 3/5 (60.0%) | 1/1 (100.0%) | 12/24 (50.0%) | 6/7 (85.7%) | 1/1 (100.0%) | 0.525  1.400 (0.495-3.956)  (0.444-4.641) | 0.735  4.000 (0.250-63.950)  - | - |
| **Low birthweight (%)** | 22/72 (30.6) | 3/10 (30.0%) | 1/2 (50.0%) | 20/48 (41.7%) | 11/14 (78.6%) | 0/2 (0.0%) | 0.211  1.623 (0.758-3.478)  (0.678-3.904) | **0.050^b^**  **8.556 (1.332-54.949)**  **(1.441-63.000)** | 1.000  0.500 (0.125-1.999)  - |

CPR, clinical pregnancy rate; LBR, live birth rate; PSM, propensity score matching. Values are shown as numbers (%). *p*1 represents *p* value of < 35 years patients between conventional group and sequential group; *p*2 represents *p* value of 35-39 years patients between conventional group and sequential group; *p*3 represents *p* value of ≥ 40 years patients between conventional group and sequential group. Bold values are statistically significant. “a” represents statistical power of post-hoc power analysis ≥ 0.70; “b” represents statistical power of post-hoc power analysis < 0.80.

**Supplementary table 3. Comparison of pregnancy outcomes of all patients based on previous failed cycles after PSM.**

|  | **Conventional group**  **(n = 272)** | | | **Sequential group**  **(n = 272)** | | |  |  |  |
| --- | --- | --- | --- | --- | --- | --- | --- | --- | --- |
| **Previous failed cycles** | **2**  **(n = 156)** | **3**  **(n = 72)** | **> 3**  **(n = 44)** | **2**  **(n = 146)** | **3**  **(n = 77)** | **> 3**  **(n = 49)** | ***p*1**  **OR (95% CI)** | ***p*2**  **OR (95% CI)** | ***p*3**  **OR (95% CI)** |
| **Biochemical pregnancy loss rate (%)** | 25 (16.0%) | 6  (8.3%) | 9 (20.5%) | 21  (14.4%) | 8 (10.4%) | 6  (12.2%) | 0.691  0.880 (0.469-1.653)  (0.448-1.696) | 0.667  1.275 (0.420-3.874)  (0.337-5.412) | 0.282  0.543 (0.176-1.672)  (0.136-1.758) |
| **CPR (%)** | 89 (57.1%) | 41  (56.9) | 18 (40.9%) | 93  (63.7%) | 54 (70.1%) | 31 (63.3%) | 0.238  1.321 (0.831-2.099)  (0.818-2.519) | 0.094^b^  1.775 (0.904-3.487)  (0.881-3.806) | **0.031^b^**  **2.488 (1.079-5.738)**  **(1.021-6.680)** |
| **Implantation rate (%)** | 129/312 (41.3%) | 60/144 (41.7%) | 26/88 (29.5%) | 124/292 (42.5%) | 70/154 (45.5%) | 40/98 (40.8%) | 0.780  1.047 (0.758-1.447)  (0.756-1.464) | 0.510  1.167 (0.737-1.846)  (0.729-1.881) | 0.109  1.645 (0.894-3.026)  (0.880-3.115) |
| **Multiple pregnancy rate (%)** | 40/89 (44.9%) | 20/41 (48.8%) | 8/18 (44.4%) | 31/93 (33.3%) | 15/54 (27.8%) | 9/31 (29.0%) | 0.108  0.613 (0.336-1.116)  (0.326-1.111) | **0.036^b^**  **0.404 (0.172-0.949)**  **(0.162-0.938)** | 0.275  0.511 (0.152-1.716)  (0.123-1.970) |
| **Ectopic pregnancy rate (%)** | 1/89 (1.1%) | 0/41 (0.0%) | 0/18 (0.0%) | 1/93 (1.1%) | 0/54 (0.0%) | 0/31 (0.0%) | 1.000  0.957 (0.059-15.529)  (0.276-3.302) | - | - |
| **Early miscarriage rate (%)** | 10/89 (11.2%) | 8/41 (19.5%) | 5/18 (27.8%) | 19/93 (20.4%) | 9/54 (16.7%) | 6/31 (19.4%) | 0.090^b^  2.028 (0.886-4.646)  (0.834-5.955) | 0.720  0.825 (0.288-2.365)  (0.255-2.962) | 0.744  0.624 (0.160-2.438)  (0.132-2.964) |
| **LBR (%)** | 77 (49.4%) | 30 (41.7%) | 13 (29.5%) | 68  (46.6%) | 45 (58.4%) | 24 (49.0%) | 0.628  0.894 (0.569-1.405)  (0.566-1.410) | **0.041^b^**  **1.969 (1.026-3.778)**  **(1.020-4.035)** | 0.056 ^b^  2.289 (0.972-5.389)  (0.931-6.476) |
| **Singleton** | | | | | | | | | |
| **Preterm birth rate (%)** | 4/51 (7.8%) | 1/19 (5.3%) | 2/8 (25.0%) | 5/51 (9.8%) | 4/36 (11.1%) | 1/18 (5.6%) | 1.000  1.277 (0.323-5.057)  (0.273-6.246) | 0.823  2.250 (0.233-21.694)  - | 0.215  0.176 (0.013-2.316)  - |
| **Low birthweight (%)** | 2/51 (3.9%) | 0/19 (0.0%) | 0/8 (0.0%) | 2/51 (3.9%) | 2/36 (5.6%) | 1/18 (5.6%) | 1.000  1.000 (0.135-7.386)  (0.221-4.571) | 0.539  1.059 (0.978-1.146)  - | 1.000  1.059 (0.947-1.184)  - |
| **Twin** | | | | | | | | | |
| **Preterm birth rate (%)** | 11/26 (42.3%) | 4/11 (36.4%) | 4/5 (80.0%) | 9/17 (52.9%) | 5/9 (55.6%) | 5/6 (83.3%) | 0.494  1.534 (0.449-5.247)  (0.379-6.600) | 0.684  2.188 (0.362-13.226)  (0.286-16.333) | 1.000  1.250 (0.058-26.869)  - |
| **Low birthweight (%)** | 14/52 (26.9%) | 6/22 (27.3%) | 6/10 (60.0%) | 13/34 (38.2%) | 8/18 (44.4%) | 10/12 (83.3%) | 0.269  1.680 (0.667-4.233)  (0.563-5.145) | 0.257  2.133 (0.569-7.993)  (0.438-13.000) | 0.458  3.333 (0.462-24.052)  - |

CPR, clinical pregnancy rate; LBR, live birth rate; PSM, propensity score matching. Values are shown as numbers (%). *p*1 represents *p* value of patients with 2 previous failed cycles between conventional group and sequential group; *p*2 represents *p* value of patients with 3 previous failed cycles between conventional group and sequential group; *p*3 represents *p* value of patients with > 3 previous failed cycles between conventional group and sequential group. Bold values are statistically significant. “b” represents statistical power of post-hoc power analysis < 0.80.

**Supplementary table 4. Comparison of pregnancy outcomes of all patients based on embryo quality after PSM.**

|  | **Conventional group (n = 272)** | | | **Sequential group (n = 272)** | | |  |  |  |
| --- | --- | --- | --- | --- | --- | --- | --- | --- | --- |
| **High-quality embryos** | **0**  **(n = 113)** | **1**  **(n = 93)** | **2**  **(n = 66)** | **0**  **(n =101)** | **1**  **(n = 103)** | **2**  **(n = 68)** | ***p*1**  **OR (95% CI)** | ***p*2**  **OR (95% CI)** | ***p*3**  **OR (95% CI)** |
| **Biochemical pregnancy loss rate (%)** | 23  (20.4%) | 13 (14.0%) | 4  (6.1%) | 18 (17.8%) | 11  (10.7%) | 6  (8.8%) | 0.638  0.849 (0.428-1.684)  (0.404-1.717) | 0.482  0.736 (0.312-1.734)  (0.272-1.846) | 0.780  1.500 (0.403-5.577)  (0.349-7.421) |
| **CPR (%)** | 45  (39.8%) | 50 (53.8%) | 53  (80.3%) | 55 (54.5%) | 69 (67.0%) | 54 (79.4%) | **0.032^b^**  **1.807 (1.049-3.111)**  **(1.036-3.162)** | 0.058^b^  1.745 (0.979-3.113)  (0.950-3.289) | 0.898  0.946 (0.407-2.202)  (0.370-2.333) |
| **Implantation rate (%)** | 57/226 (25.2%) | 70/186 (37.6%) | 88/132 (66.7%) | 70/202 (34.7%) | 90/206 (43.7%) | 74/136 (54.4%) | **0.033^b^**  **1.572 (1.036-2.386)**  **(1.030-2.423)** | 0.223  1.286 (0.858-1.927)  (0.849-1.962) | **0.040^b^**  **0.597 (0.364-0.979)**  **(0.355-0.955)** |
| **Multiple pregnancy rate (%)** | 13/45 (28.9%) | 20/50 (40.0%) | 35/53 (66.0%) | 15/55 (27.3%) | 20/69 (29.0%) | 20/54 (37.0%) | 0.858  0.923 (0.384-2.217)  (0.338-2.538) | 0.209  0.612 (0.284-1.320)  (0.267-1.350) | **0.003^a^**  **0.303 (0.137-0.668)**  **(0.128-0.628)** |
| **Ectopic pregnancy rate (%)** | 0/45  (0.0%) | 1/50 (2.0%) | 0/53 (0.0%) | 0/55 (0.0%) | 0/69 (0.0%) | 1/54 (1.9%) | - | 0.420  0.980 (0.942-1.020)  - | 1.000  1.019 (0.982-1.057)  - |
| **Early miscarriage rate (%)** | 7/45  (15.6%) | 9/50 (18.0%) | 7/53 (13.2%) | 11/55 (20.0%) | 16/69 (23.2%) | 7/54 (13.0%) | 0.565  1.357 (0.479-3.849)  (0.461-4.609) | 0.493  1.375 (0.552-3.426)  (0.520-4.302) | 0.970  0.979 (0.318-3.011)  (0.249-4.107) |
| **LBR (%)** | 36  (31.9%) | 39 (41.9%) | 45  (68.2%) | 42 (41.6%) | 51 (49.5%) | 44 (64.7%) | 0.140  1.523 (0.870-2.665)  (0.859-2.686) | 0.288  1.358 (0.772-2.388)  (0.759-2.510) | 0.670  0.856 (0.417-1.754)  (0.397-1.783) |
| **Singleton** | | | | | | | | | |
| **Preterm birth rate (%)** | 1/30  (3.3%) | 3/25 (12.0%) | 3/23 (13.0%) | 1/33 (3.0%) | 5/40 (12.5%) | 4/32 (12.5%) | 1.000  0.906 (0.054-15.158)  (0.237-3.400) | 1.000  1.048 (0.227-4.826)  - | 1.000  0.952 (0.192-4.732)  (0.167-4.756) |
| **Low birthweight (%)** | 1/30  (3.3%) | 0/25 (0.0%) | 1/23 (4.3%) | 1/33 (3.0%) | 2/40 (5.0%) | 2/32 (6.3%) | 1.000  0.906 (0.054-15.158)  (0.235-3.237) | 0.519  1.053 (0.980-1.130)  - | 1.000  1.467 (0.125-17.213)  (0.222-3.962) |
| **Twin** | | | | | | | | | |
| **Preterm birth rate (%)** | 4/6  (66.7%) | 3/14 (21.4%) | 12/22 (54.5%) | 8/9 (88.9%) | 5/11 (45.5%) | 6/12 (50.0%) | 0.693  4.000 (0.273-58.562)  (0.429-20.000) | 0.397  3.056 (0.535-17.462)  (0.500-21.000) | 0.800  0.833 (0.204-3.409)  (0.167-4.125) |
| **Low birthweight (%)** | 6/12  (50.0%) | 7/28 (25.0%) | 13/44 (29.5%) | 10/18 (55.6%) | 10/22 (45.5%) | 11/24 (45.8%) | 0.765  1.250 (0.289-5.407)  (0.238-6.933) | 0.130  2.500 (0.754-8.286)  (0.647-12.833) | 0.179  2.018 (0.719-5.661)  (0.615-6.878) |

CPR, clinical pregnancy rate; LBR, live birth rate; PSM, propensity score matching. Values are shown as numbers (%). *p*1 represents *p* value of patients with 0 high-quality embryo between conventional group and sequential group; *p*2 represents *p* value of patients with 1 high-quality embryo between conventional group and sequential group; *p*3 represents *p* value of patients with 2 high-quality embryos between conventional group and sequential group. Bold values are statistically significant. “a” represents statistical power of post-hoc power analysis ≥ 0.70; “b” represents statistical power of post-hoc power analysis < 0.80.

**Supplementary figure 1. Standardized mean differences for baseline covariates comparing sequential group to conventional group in the original and the matched sample.**

AMH, anti-Müllerian hormone; bFSH, basal follicle-stimulating hormone; BMI, body mass index.
